# Pten and p53 Loss in the Mouse Lung Causes Adenocarcinoma and Sarcomatoid Carcinoma

**DOI:** 10.3390/cancers14153671

**Published:** 2022-07-28

**Authors:** Sara Lázaro, Corina Lorz, Ana Belén Enguita, Iván Seller, Jesús M. Paramio, Mirentxu Santos

**Affiliations:** 1 Molecular Oncology Unit, Centro de Investigaciones Energéticas, Medioambientales y Tecnológicas (CIEMAT), Ave Complutense 40, 28040 Madrid, Spain; sara.lazaroe@gmail.com (S.L.); clorz@ciemat.es (C.L.); ivan.seller@ciemat.es (I.S.); jesusm.paramio@ciemat.es (J.M.P.); 2 CIBERONC—Centro de Investigación Biomédica en Red de Cáncer, 28029 Madrid, Spain; 3 Institute of Biomedical Research Hospital “12 de Octubre” (imas12), Ave Córdoba s/n, 28041 Madrid, Spain; 4 Pathology Department, University Hospital “12 de Octubre”, 28041 Madrid, Spain; abenguita.hdoc@salud.madrid.org

**Keywords:** PTEN, p53, lung adenocarcinoma, pulmonary sarcomatoid carcinoma

## Abstract

**Simple Summary:**

Lung cancer is the world leading cause of cancer death. Therefore, a better understanding of the disease is needed to improve patient survival. In this work, we have deleted the tumor suppressor genes *Pten* and *Trp53* in adult mouse lungs to analyze its impact on tumor formation. Double mutant mice develop Adenocarcinoma and Pulmonary Sarcomatoid Carcinoma, two different types of Non-Small Cell Carcinoma whose biological relationships are a matter of debate. The former is very common, with various models described and some therapeutic options. The latter is very rare with very poor prognosis, no effective treatment and lack of models reported so far. Interestingly, this study reports the first mouse model of pulmonary sarcomatoid carcinoma available for preclinical research.

**Abstract:**

Lung cancer remains the leading cause of cancer deaths worldwide. Among the Non-Small Cell Carcinoma (NSCLC) category, Adenocarcinoma (ADC) represents the most common type, with different reported driver mutations, a bunch of models described and therapeutic options. Meanwhile, Pulmonary Sarcomatoid Carcinoma (PSC) is one of the rarest, with very poor outcomes, scarce availability of patient material, no effective therapies and no models available for preclinical research. Here, we describe that the combined deletion of *Pten* and *Trp53* in the lungs of adult conditional mice leads to the development of both ADC and PSC irrespective of the lung targeted cell type after naphthalene induced airway epithelial regeneration. Although this model shows long latency periods and incomplete penetrance for tumor development, it is the first PSC mouse model reported so far, and sheds light on the relationships between ADC and PSC and their cells of origin. Moreover, human ADC show strong transcriptomic similarities to the mouse PSC, providing a link between both tumor types and the human ADC.

## 1. Introduction

Lung cancer remains the deadliest cancer condition worldwide [1]. Thus, improving patient survival is an unmet need, which emphasizes the absolute requirement to extend our current comprehension of the underlying mechanisms of the disease. Lung Adenocarcinoma (ADC) is the most common primary lung cancer seen and represents about 40% of all lung cancers [2]. It usually evolves from the mucosal glands. When feasible, complete tumoral resection is considered the best treatment option; however, targeted therapies (mainly tyrosine kinase inhibitors) and immunotherapies may induce clinical responses in suitable patient subgroups [3]. In sharp contrast to adenocarcinoma (ADC), Pulmonary Sarcomatoid Carcinoma (PSC) is a rare (0.3% to 1% of all pulmonary malignancies) category of highly aggressive and poorly differentiated tumors with poor prognosis and few treatment options, as patients show limited response or resistance to conventional chemotherapy [4,5,6]. They are both gathered together under the umbrella of the non-small cell lung carcinoma (NSCLC) group. In the last few years, an effort has been made in the molecular characterization of PSC and the development of targeted therapies, such as the identification of mutations in the MET gene, and the reported high expression of PD-L1 [7,8,9]. Nevertheless, no standard treatment is currently available, and owing to its low prevalence there is a remarkable scarcity of material from human patients, which makes the development of preclinical models of crucial importance and an urgent unmet need.

*TP53* alterations are a frequent characteristic of all lung cancer types. Loss or mutation of p53 is known to cause global genetic instability and to promote additional pro-tumorigenic mutations due to its critical role in regulating DNA repair, cell cycle, programmed cell death, and cellular senescence [10]. *TP53* appears to play a particularly important role in PSC. It is the most frequently mutated gene in this condition (74–79%) and most of the reported mutations lead to gene inactivation [8,11,12]. *TP53* mutations are also found with high frequency (50–52%) in lung ADC [13,14]. In addition to the p53 pathway, the PI3K pathway is commonly dysregulated in human cancer. Phosphatase and tensin homolog (PTEN) is a well-characterized tumor suppressor protein involved in the negative regulation of the phosphoinositide 3-kinase (PI3K) pro-growth pathway. PTEN lies upstream of the phosphoinositide 3-kinase (PI3K)-AKT-mammalian target of the rapamycin (mTOR) axis, which has broad roles in directing cellular growth, metabolism, division, senescence, and migration [15]. Loss of PTEN expression has been described in approximately 9% in PSC [11] and occurs in 5–10% of ADC [14,16,17].

The impact of the combined inactivation of these tumor suppressors, a frequent event in squamous cell lung cancer [18], is poorly understood. Genetically Engineered Mouse Models (GEMMs) of lung cancer provide an excellent tool to dissect mechanisms responsible for the appearance and progression of the disease and to identify cells of origin [19,20,21,22,23]. To gain a deeper insight into the combined role of *PTEN* and *TP53* deletions in lung cancer, we have generated mouse models in which both *Pten* and *Trp53* are disrupted in lung epithelial cells, either widely (by means of an Ad5-CMVcre virus) or in a cell-type restricted manner (using an Ad5-K5cre virus) and determine its effect on tumor formation. The simultaneous deletion of *Pten* and *Trp53* in sporadic cells of the lung results in the development of ADC and PSC with low penetrance and long latency periods. This is irrespective of the targeted initiating cell in the adult lung, at least in naphthalene treated mice. Naphthalene induced injury and epithelial airway regeneration, accelerates tumor development to some extent and favors the development of PSC. Akt pathway activation and epithelial–mesenchymal transition (EMT) have been analyzed in double-deficient tumors, with clear signs of EMT observed in PSC. Moreover, both mouse ADC and PSC show transcriptomic similarities to the human ADC reported data. As far as we know, no models for PSC, a particularly interesting tumor to study, have been described so far. Owing to its rarity, there is a notable paucity of biologic material for the study of PSC, which has hampered significant advances in patient treatment, currently in search for an effective therapy. Thus, given the lack of models for this disease, the development of a model for PSC is prominent, as it provides a valuable tool for the comprehension of the disease and the development of novel therapeutic approaches.

## 2. Results

### 2.1. Combined Deletion of Pten and p53 in Epithelial Cells of Adult Lungs

**Figure 1:**
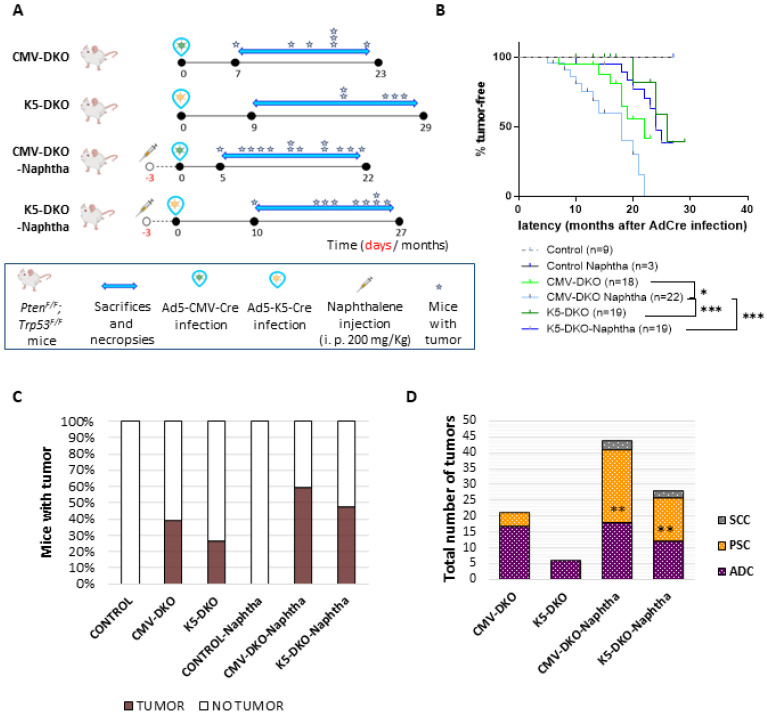
**Combined deletion of *Pten* and *p53* in adult lungs.** (**A**) Schematic of the mouse model experiment. Time course of naphthalene administration, adenovirus injection and mouse necropsy. (**B**) Kaplan–Meier tumor-free survival curve of *Trp53^F/F^*; *Pten^F/F^* control mice (dashed grey line *n* = 9), mice treated with 200 mg/kg naphthalene (black *n* = 3) infected with Ad-CMVcre virus (pale blue *n* = 18), Ad5-K5cre virus (pale green *n* = 19) and administered with 200 mg/kg naphthalene and infected with Ad5-CMVcre virus (dark blue *n* = 22) or Ad5-K5cre virus (dark green *n* = 19). The median tumor-free survival of the CMV-DKO mice and K5-DKO is 22 and 26 months, respectively, and for CMV-DKO and K5-DKO mice treated with naphthalene prior to infection with adenovirus, tumor-free survival is 18 and 24 months, respectively. * *p* < 0.05; *** *p* ≤ 0.0001 determined by log-Rank. (**C**) Incidence of tumors in uninfected *Trp53^F/F^*; *Pten^F/F^* mice with or without naphthalene injection and DKO mice after adenovirus infection with Ad5-CMVcre or Ad5-K5cre with or without naphthalene injection. Total number of mice: Control *n* = 9; Control Naphtha *n* = 3; CMV-DKO *n* = 18; CMV-DKO Naphtha *n* = 22; K5-DKO *n* = 19; K5-DKO Naphtha *n* = 22. (**D**) Histopathology spectrum of tumors arisen from *Trp53^F/F^*; *Pten^F/F^* mice infected with Ad5-CMVcre virus (CMV-DKO) or Ad5-K5cre virus (K5-DKO) and after naphthalene injection (CMV-DKO Naphtha; K5-DKO Naphtha). Total number of tumors CMV-DKO *n* = 21; CMV-DKO Naphtha *n* = 44; K5-DKO *n*= 6; K5-DKO Naphtha *n* = 28. ADC: adenocarcinoma; PSC: pulmonary sarcomatoid carcinoma; SCC: squamous cell carcinoma; ** *p* ≤ 0.001 determined by Fischer exact *t*-test.

As previously shown [24,25], we have taken advantage of the CMV promoter (Ad5-CMVcre, which targets all types of epithelial cells) and the K5 promoter (Ad5-K5cre, which specifically targets basal cells) to interrogate the role of the combined deletion of the tumor suppressors *Pten* and *Trp53* in adult lungs. To do this, we have generated the CMV-DKO and K5-DKO mice by intratracheal infection of the adenovirus Ad5-CMVcre and Ad5-K5cre, respectively, in the *Trp53^F/F^*; *Pten^F/F^* mice (Figure 1A).

Mice were sacrificed when they showed signs of disease (which include weight loss, shortness of breath, lethargy, hunched posture or ruffled fur) and necropsy was performed (Figure S1). CMV-DKO mice (*n* = 18) developed tumors with a latency of 7–22 months and a frequency of 39% (Figure 1A–C, Tables S1 and S2). For the K5-DKO group (*n* = 19), the latency period was 20–26 months and the incidence was 26%. Nine uninfected *Trp53^F/F^*; *Pten^F/F^* littermates were followed up as controls and did not develop any sign of illness up to 26 months. Another three additional mice were treated with naphthalene (control + naphtha) and followed up without developing tumors or showing histological respiratory injury (Figure 1B,C).

We treated DKO mice with naphthalene to explore the effects of naphthalene-induced lung injury on DKO-tumor development. Naphthalene-induced lung injury ablates Clara Cells (Figure S2), exposes airways basal cells to external agents, increases Keratin 5 expressing tracheal basal cells (Figure S2), is accompanied by proliferation of the principal lung cell types [26,27] and has been related to development of lung tumors [28]. DKO mice were pretreated with naphthalene and intratracheally injected with either Ad5-CMVcre (*n* = 22) or Ad5-K5cre (*n* = 19) to initiate tumorigenesis three days after lung injury. Three *Trp53^F/F^*; *Pten^F/F^* mice were treated only with naphthalene. Infected mice developed tumors with a latency of 5–22 months (CMV-DKO + Naphtha) or 10–25 months (K5-DKO + Naphtha) and an incidence of 59% or 47%, respectively (Figure 1B,C).

Wide targeting of lung epithelial cells (by using Ad5-CMVcre) rendered a higher percentage of mice with tumors and reduced latency periods with respect to basal cell restricted targeting (Ad5-K5cre) of *Pten* and *Trp53*. DKO mice showed a higher incidence in the percentage of tumor-bearing mice depending whether or not naphthalene had been administered prior to adenovirus infection and irrespective of the targeted cell/adenovirus used (Figure 1B,C). Ad5-CMVcre (but not Ad5-K5cre)-infected mice showed a significant difference in tumor-free survival depending upon naphthalene administration (Figure 1B). Thus, naphthalene increased overall incidence of mice with lung tumors and accelerated tumor development after wide targeting loss of *Pten* and *Trp53* in lung epithelial cells.

### 2.2. Development of Adenocarcinoma and Pulmonary Sarcomatoid Carcinoma in Double-Deficient Lungs

**Figure 2:**
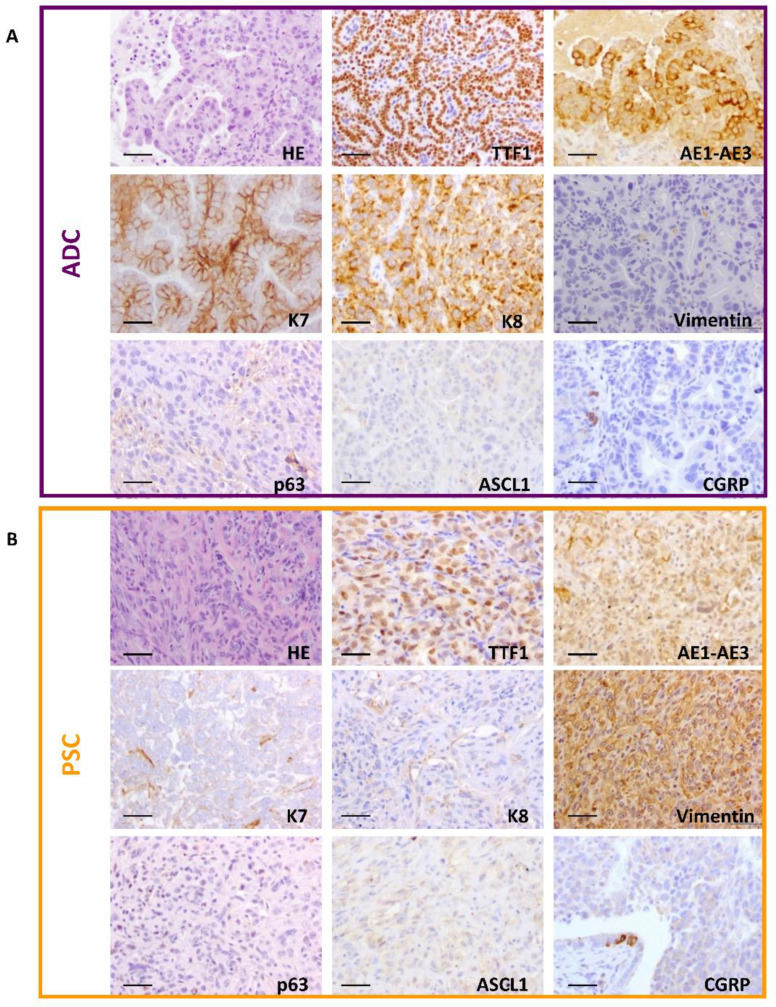
**Adenocarcinoma and pulmonary sarcomatoid carcinoma developed in double-deficient lungs.** Representative hematoxylin eosin staining of ADC (**A**) and PSC (**B**) lung tumors of DKO mice. Immunohistochemical analysis of lung tumors with the quoted antibodies. Mouse ADCs were positive for thyroid transcription factor-1 (TTF-1), keratin K7 and K8 and pan cytokeratin AE1–AE3, and negative for vimentin. PSCs showed positive immunostaining for TTF-1 and vimentin, weak signal for AE1–AE3 and negative staining for keratin K7 and K8. Tumors were negative for the epithelial marker p63 and the neuroendocrine markers ASCL1 and CGRP. Bars = 50 µm.

Histopathological analyses of CMV-DKO and K5-DKO tumors led to the diagnosis of two main lung tumor types: ADC and PSC (Figure 2, Tables S1 and S2). Occasionally, lung squamous cell carcinoma was observed (Tables S1 and S2, Figure S3), and this was the primary tumor type for two Ad5-CMVcre-infected mice. Adenocarcinomas displayed glandular differentiation with characteristic acinar, papillary or lepidic patterns (Figure S3) [29]. PSCs showed the histological heterogeneity described in human patients [30] with pleomorphic, spindle, and giant cell carcinoma and carcinosarcoma variants were observed (Figure S3). These histologic subtypes are useful for the recognition and pathology diagnoses of PSC, but they do not seem to have clinical or therapeutic value [4,31,32]. To our knowledge, this is the first time a pulmonary sarcomatoid carcinoma is reported in a mouse model.

**Figure 3:**
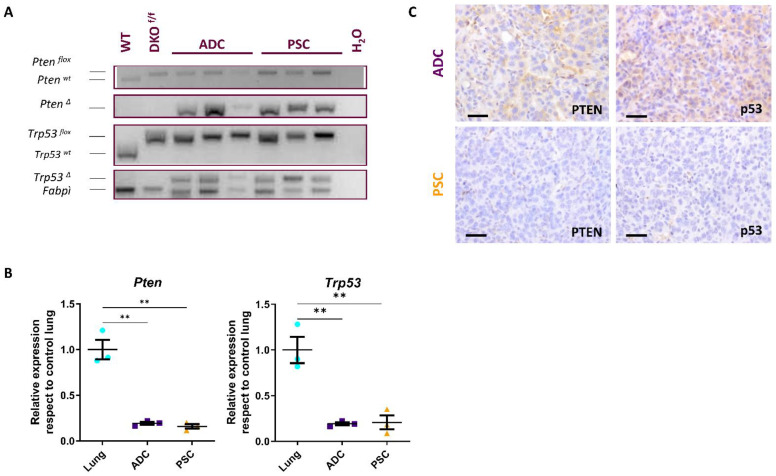
**Deletion of *Trp53* and *Pten* in adenocarcinoma (ADC) and pulmonary sarcomatoid carcinoma (PSC) arisen in CMV-DKO and K5-DKO mice.** (**A**) PCR analysis of *Trp53* and *Pten* floxed genes confirms the deletion in double mutant tumors of the *Pten* and *Trp53* gene. WT: wild type mouse lung, DKO^F/F^: uninfected *Trp53^F/F^*; *Pten^F/F^* mouse lung. *Pten^w^*^t^ and *Trp53^wt^*: *Pten* and *Trp53* wild type alleles. *Pten^flox^* and *Trp53^flox^*: *Pten* and *Trp53* floxed alleles. *Pten^∆^* and *Trp53^∆^*: *Pten* and *Trp53* deleted alleles. *Fabpi*: loading control. Original image of PCR can be found at File S1. (**B**) qRT-PCR analysis of *Trp53* and *Pten* expression levels in double mutant tumors (ADC and PSC) and control lungs (lung *n* = 3; ADC *n* = 3; PSC *n* = 3). ** *p* < 0.01, determined by *t*-test. *Tbp* was used as a housekeeping gene. (**C**) Immunohistochemical analyses of PTEN and TP53 showing the absence of protein staining in ADC and PSC. Bars = 50 µm.

Deletion of both *Pten^F/F^* and *Trp53^F/F^* alleles in these tumors was observed by PCR (Figure 3A), further confirmed by RT-qPCR (Figure 3B), and consequently, no detection of the corresponding proteins PTEN and p53 was observed by immunohistochemistry (Figure 3C). Tumors were further characterized by immunohistochemical staining (Figure 2). The *Pten*/*Trp53*-deficient ADCs expressed thyroid transcription factor1 (TTF-1), and Keratins K7 and K8 showed positive staining for pan-cytokeratin AE1–AE3 and were negative for the mesenchymal marker vimentin. Meanwhile, pulmonary sarcomatoid tumors developed upon inactivation of *Pten* and *Trp53* expressed TTF-1 and vimentin, displaying weak staining of cytokeratins using the AE1–AE3 antibody and absence of keratins K7 and K8. Both tumors were negative for the epithelial marker p63 (marker for squamous cell carcinoma) and the neuroendocrine markers calcitonin gene related protein (CGRP) and Achaete-scute complex homolog-1 (MASH1/ASCL1), characteristic of neuroendocrine tumors (Figure 2).

There was no difference between the tumor type developed regarding the adenovirus injected: both ADC and PSC arose from basal cells (Ad5K5cre-DKO) as well as from a variety of epithelial lung cells (Ad5CMVcre-DKO), such as Clara, Alveolar Type 1 and alveolar Type 2 cells [24,25], suggesting multiple cells of origin for both ADC and PSC. We observed a clear overlap in the histopathological features in the lungs following either Ad5-CMVcre or Ad5-K5cre administration only after naphthalene administration (Figure 1D). DKO mice treated with naphthalene (irrespective of the adenovirus used to initiate tumorigenesis) had a higher incidence of PSCs carcinomas compared to non-treated mice (Figure 1D). The difference was found to be significant by a Fischer exact *t*-test, *p* = 0.001. Thus, naphthalene treatment favors the development of PSC over ADC in double-deficient lungs.

Metastatic lesions were present in 18% of Ad5-CMVcre and Ad5-K5cre naphthalene treated bearing-tumor mice (4 mice out of 22). Primary tumors in these mice (2 ADC and 4 PSC), were able to colonize distant organs, including the liver, and develop PSC. Metastatic lesions displayed histological features and vimentin expression characteristic of PSC (Figure S4). This observation highlights the ability of *Pten*/*Trp53* deficient mice after treatment with naphthalene to recapitulate the behavior of human PSC, which show metastasis to distal organs [33,34].

### 2.3. Akt Pathway Activation and Epithelial–Mesenchymal Transition (EMT) Occur in Pten and Trp53 Double-Deficient Mouse Tumors

**Figure 4:**
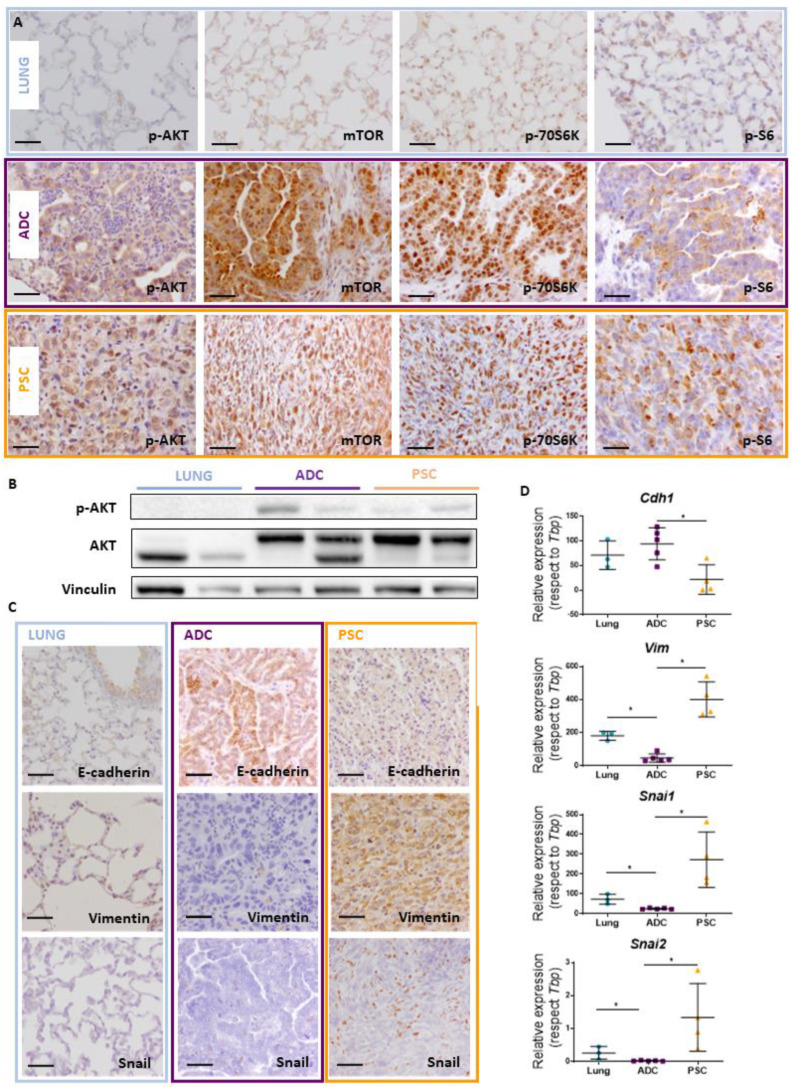
**Akt pathway activity and EMT process in adenocarcinoma (ADC) and pulmonary sarcomatoid carcinoma (PSC) arisen in DKO mice**. (**A**) Sections from lungs (upper panel), ADC (middle panel) and PSC (lower panel) were immunostained for p-Akt, mTOR, p70S6K and pS6, as indicated. Bars = 50 µm. (**B**) p-AKT and AKT were assessed using Western blotting analyses. Original image of western blot can be found at File S1 (**C**) Immunohistochemical analyses for the quoted proteins (E-cadherin, Vimentin and Snail) involved in the EMT processes in lungs (left column), ADC (middle column) and PSC (right column). Bars = 50 µm. (**D**) qRT-PCR analysis of *Cdh1*, *Vim*, *Snai1* and *Snai2* (encoding for E-cadherin, Vimentin, Snail and Slug, respectively) expression levels in ADC, PSC and control lungs. * *p* < 0.05, determined by Mann–Whitney.

We assessed the impact of *Trp53*/*Pten* deletion on Akt signaling. As expected for Pten-deficient tumors, Akt-pathway activity was elevated in both ADC and PSC *Trp53*/*Pten* double-mutant tumors (Figure 4A). Consistently, p-Akt, mTOR, p70S6K and pS6 proteins were clearly detected in histological sections of the tumors, while control lungs were negative for Akt-P and showed weak expression of mTOR, p70S6K and pS6 proteins in pneumocytes (Figure 4A). An increase in Akt and p-Akt proteins in tumors was further confirmed by Western blot (Figure 4B).

Given the characteristics observed in the tumors obtained and as EMT processes have been involved in the carcinogenic mechanisms and evolution of PSC [35,36,37], we tested the expression of EMT transcription factors in lungs, ADCs and PSCs (Figure 4D). The hallmark epithelial marker E-cadherin increased in ADCs while it decreased in PSCs. Opposite to this finding and consistent with an EMT process, Vimentin, Snai1 and Snai2 mRNA levels were decreased in ADCs and increased in PSCs. Similar to the mRNA levels, E-cadherin protein was seen in lung epithelial cells (mainly bronchiole) and detected in adenocarcinomas, while vimentin (positive in myoepithelial cells of control lungs) and snail proteins (negative in lung) were detected in PSC tumors by Immunohistochemistry (Figure 4C). Thus, PSCs exhibit characteristics of EMT, including a reduction of E-cadherin and an increase of Vimentin, Snail and Slug expression, supporting that PSCs have undergone EMT.

### 2.4. Transcriptomic Analysis of Double-Deficient Pten and Trp53 Mouse Lung Tumors

**Figure 5:**
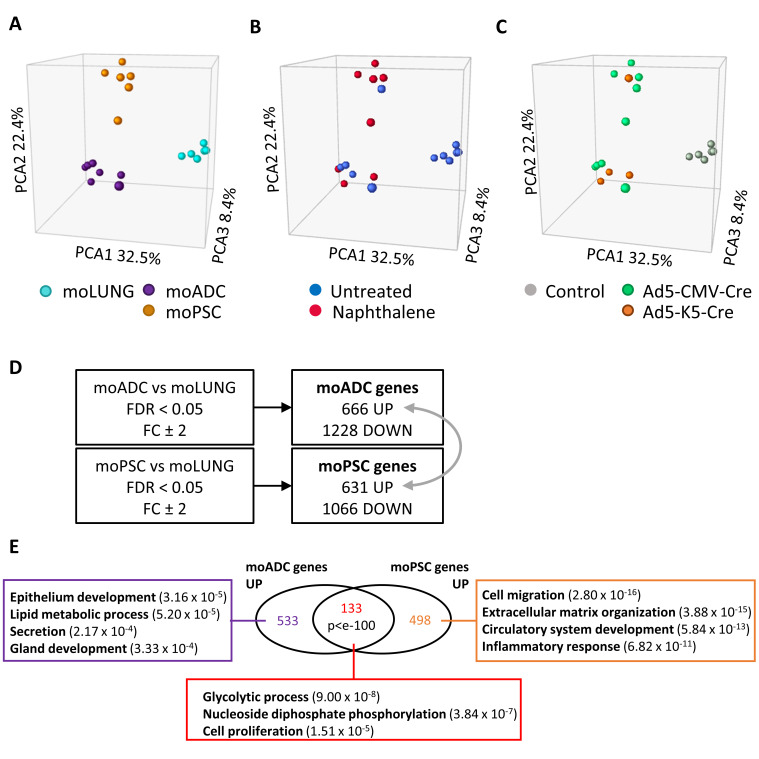
**Gene expression analysis of lung ADC and PSC arising from DKO mice.** Principal component analysis (PCA) plots showing the distribution of the samples along the PC1, PC2 and PC3 axes and labeled according to: (**A**) their histology type (moLUNG, pale blue color; moADC, purple; moPSC, orange); (**B**) untreated (blue) or naphthalene treated (red) samples; (**C**) the adenoviral vector used (control uninfected, grey; Ad5-CMVcre, green; Ad5-K5cre, orange). (**D**) Genes significantly (FDR < 0.05) upregulated or downregulated more than two-fold in moADC or in moPSC compared to moLUNG. Numbers indicate Affymetrix probe set identifiers. FDR, false discovery rate; FC, fold change. (**E**) Gene ontology analysis of moADC and moPSC upregulated genes (grey arrow in (**D**)). The Venn diagram shows genes common to both tumor types (133) or specific of moADC (533) or moPSC (498). Hypergeometric test was used to assess the statistical significance of the overlap (*p* < 10^−100^). The rectangular boxes contain the main signaling pathways enriched in the indicated groups (gene ontology biological processes). *p*-values in brackets.

To further characterize these lung tumors, we performed microarray analysis of both tumor types, mouse ADC (moADC, *n* = 7) and mouse PSC (moPSC, *n* = 6), from mice treated with both adenoviral vectors and with/without naphthalene, and lung tissue from untreated and uninfected littermates (moLUNG, *n* = 6) (Tables S3 and S4). The principal component analysis (PCA) grouped the samples into three sets, according to the histopathologic sample type (i.e., lung, ADC and PSC) (Figure 5A). PSC was the group that displayed the highest intragroup heterogeneity. Naphthalene treatment or the initial targeted cell type did not seem to have an impact in the transcriptional characteristics of the tumors (Figure 5B,C).

We compared the gene expression profiles of each tumor type with normal lung, to select for genes that were specifically deregulated in PSC or ADC (Figure 5D). Next, we analyzed the enrichment in molecular pathways in the upregulated genes that were either shared or distinctive of moADC or moPSC. Gene ontology pathways significantly enriched in the upregulated genes common to moADC and moPSC were *glycolytic processes*, *nucleoside diphosphate phosphorylation* and *cell proliferation*, indicating higher metabolic activity and proliferation of these tumors versus normal lung (Figure 5E; Table S5). In agreement, Gene Set Enrichment Analysis (GSEA) showed that both tumor types were enriched in *glycolysis*, *E2F targets* and *G2M checkpoint* hallmark signatures (Molecular Signatures Database, MSigDB, hallmark gene sets) compared to lung (Figure S5A). Genes specifically upregulated in moADCs were consistent with a glandular phenotype, while genes specifically upregulated in moPSC revealed a more aggressive/undifferentiated phenotype (Figure 5E). In fact, when we directly compared moADC with moPSC using GSEA, moPSC were significantly enriched in hallmark gene sets of *epithelial–mesenchymal transition*, *inflammatory response*, *angiogenesis* and *interferon-gamma response* and depleted in *fatty acid metabolism* genes (Figure S5B).

**Figure 6:**
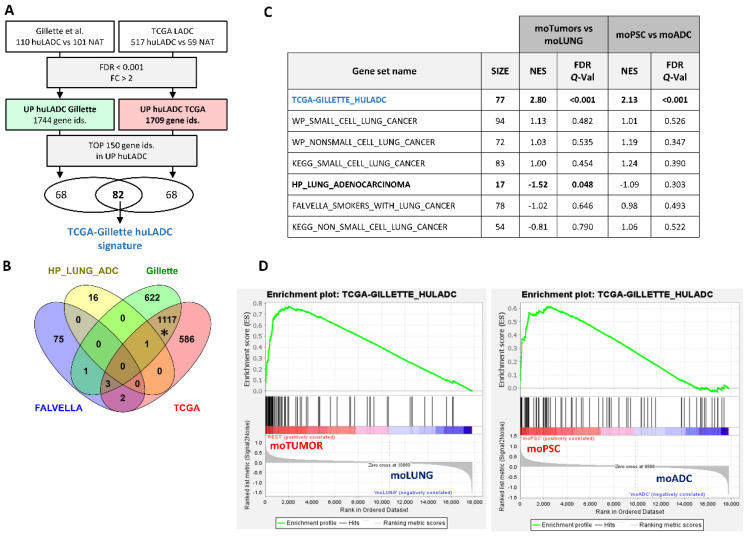
**Gene expression studies of a human lung ADC signature on mouse ADC and PSC tumors**. (**A**) Flow diagram followed to obtain a human LADC gene signature based on the Gillette et al. and the TCGA lung adenocarcinoma RNAseq data. Numbers indicate Affymetrix probe set identifiers. (**B**) Venn diagram [39] showing the overlap among TCGA, Gillette and MSigDB adenocarcinoma gene signatures (FALVELLA and HP_LUNG_ADENOCARCINOMA). Hypergeometric test was used to assess the statistical significance of the overlap: * *p* < 0.0001, representation factor 7.5; the rest *p* > 0.05. NAT: normal adjacent tissue; huADC: human lung adenocarcinoma. (**C**) Gene enrichment analysis of the indicated gene sets in the mouse tumors compared to control lung (moTumors vs. moLUNG), and in PSC compared to ADC mouse tumors (moPSC vs. moADC). Significant (FDR *Q*-Val < 0.25) gene sets are highlighted in bold. SIZE: number of genes in gene set. NES: Normalized Enrichment Score. The normalized enrichment score (NES) is used to compare analysis results across gene sets. (**D**) Enrichment plots for the TCGA-GILLETTE_HULADC gene set. The enrichment score (ES) reflects the degree to which the gene set is overrepresented at the top (positive ES values) or bottom (negative ES values) of the ranked list of genes in the expression dataset.

To compare the gene expression profiles of the lung tumors from our DKO mouse model with human lung adenocarcinomas (huLADC), we developed two huLADC gene signatures based on the RNAseq data available from Gillette et al. [38] and the Tumor Cancer Genome Atlas (TCGA Lung Adenocarcinoma). Gillete et al.’s study included 110 treatment-naive human LADC tumors and 101 paired normal adjacent tissue (NAT). TCGA data included 517 LADC and 59 NAT (see materials and Methods section). Within each study, huLUAD samples were compared to NAT and significantly upregulated genes were selected (Figure 6A, Table S6). We compared these two huLADC gene signatures with two other signatures for human LADC form the MSigDB, FALVELLA and HP_LUNG_ADENOCARCINOMA, to look for common genes (Figure 6B). Gillette and TCGA huLADC signatures shared > 60% of their genes; however, the overlap of the MSigDB huLADC signatures with Gillette and TCGA was less than <0.1% and was not significant.

Next, we selected the common genes within the top 150 upregulated genes in TCGA and Gillette huLADC signatures to create a TCGA-Gillete_huLADC gene signature (Figure 6A). We used this signature, the FALVELLA and HP_LUNG_ ADENOCARCINOMA, and other signatures for small cell lung cancer (SCLC) and non-small cell lung cancer (NSCLC) from the MSiGDB (Table S6, Figure S6) to analyze enrichment in the mouse tumors (moADC and moPSC) versus normal lung (moLUNG). The human LADC signature developed here, TCGA-Gillete_huLADC, but not signatures for other lung cancer types (small cell lung cancer), was significantly enriched in our mouse tumors, indicating that the expression characteristics of the mouse tumors developed by the inactivation of the *Trp53* and *Pten* genes are similar to human LADC (Figure 6C). The MSigDB HP_LUNG_ ADENOCARCINOMA signature showed negative enrichment in mouse tumors. This could be partly due to the small gene size (*n* = 17) of the signature and the fact that it includes genes commonly mutated and/or deleted in lung cancer such as TP53 and KRAS. Similarly, we did not find enrichment in our mouse tumors of WP (WikiPathways) or KEGG (Kyoto Encyclopedia of Genes and Genomes) non-small cell lung cancer signatures, which include ADCs together with SCCs and large cell carcinoma. These are curated gene signatures that also include genes, such as KRAS, TP53 and RB1. As for the FALVELLA signature, it is the only one that was created using analysis of gene expression data; however, it used fewer samples (24 human LADC and 24 NAT) than TCGA-Gillette and was designed to distinguish smokers from non-smokers in addition to tumor versus non-tumor [40]. Interestingly, the TCGA-Gillette human ADC gene signature was significantly enriched in the mouse PSC tumors compared to mouse ADC tumors (Figure 6C,D), highlighting the similarity between moPSC tumors and human LADC.

## 3. Discussion

We disrupted *Pten* and *Trp53* with two different cre-deleter lines: CMVcre (which targets all types of lung epithelial cells) [24] and K5cre (which targets specifically airway basal cells) [25]. The histology, immunohistochemistry and transcriptomic analysis revealed that tumors driven by these two promoter-cre lines were indistinguishable, even after naphthalene induced lung injury. This is in contrast to our previous work in which the targeted cell initiating tumorigenesis determines the type of high-grade neuroendocrine lung tumor developed when Pten and *Trp53* along with *Rb1* and *Rbl1* are ablated [20,25]. However, it sheds light on the hypothesized multiple cells of origin of PSCs. Recently, Yang et al. [11], in their molecular characterization of a good-sized cohort of PSC patients, inferred that the cell origin of this tumor could be similar to that of adenocarcinoma, which has been reported to originate from Clara cells, alveolar epithelial cells and basal cells [13,21,41]. The work described here further supports their hypothesis as: (i) mouse models develop PSC (and ADC) arisen from basal cells (Ad5K5cre-DKO) as well as from other lung epithelial cells (Ad5-CMVcre-DKO); (ii) comparative genomics with a signature of human ADC clearly identified both mouse tumors as ADC.

PSC is a term comprising different histological subtypes with different morphology, suggesting heterogeneity [30]. Patients often exhibit tumors with combined carcinomatous and sarcomatoid components [42]. The coexistence of (well-differentiated) epithelial and (poorly differentiated) sarcomatoid cells has led to the hypothesis that PSC might represent an epithelial neoplasia undergoing divergent tissue differentiation [6,11,36]. PSC cells are likely derived from the epithelial–mesenchymal transition. The current work shows that, after combined deletion of *Pten* and *Trp53* in lung epithelial cells, the PSC tumors developed undergo an EMT process, unlike the ADC tumors (arisen when the same set of genes are deleted in the same targeted cells) that preserve their epithelial nature. In fact, analysis of the transcriptome profiles of the mouse tumors revealed that PSC show hallmark features of EMT. These data are in line with an increasing number of studies based on the hypothesis that pulmonary sarcomatoid cells may be derived from carcinoma cells through the activation of an EMT process that leads to sarcomatous transformation of the carcinoma cells [11,36,43,44]. This also highlights the potential of targeting EMT in the treatment strategies for PSC.

The adeno-cre intratracheal infection has proven to be a robust method of modeling lung cancer in mice [19,21,45]. Both Ad5-CMVcre and Ad5-K5cre viral vectors have been successfully used in the generation of lung tumor in mice [20,25,46]. Probably, Ad5-CMVcre is the most widely adenoviral vector used for this purpose [21,47]. Using this viral vector for wide targeting of lung epithelial cells, the Pten-p53 deletion gives rise to ADC and PSC proving that this combination of tumor suppressors acts as genetic drivers of both tumoral types. However, tumors develop after long latency periods with incomplete penetrance. Naphthalene treatment slightly accelerates the process, suggesting that additional mutations are needed. Lung ADCs are frequently characterized by different oncogenic driver mutations that affect a variety of kinases and their downstream signaling pathways [48,49,50,51]. In fact, both *Pten* and *Trp53* have been reported to accelerate Kras lung ADC formation [45,52,53,54]. Unfortunately, very little is known about the molecular events underlying development of PSC and the potential driver mutations characterizing this tumor. As an example, given the frequency of actionable *MET* gene mutations described for PSC patients [7,55], it could be interesting to generate a model approaching loss of *Pten* and *Trp53* along with the reported MET mutation. Other novel mutations identified as potential candidates in the molecular pathogenesis of PSC, such as *CDH4*, *CDH7*, *LAMB4*, *SCAF1* and *LMTK2* [7] are worth considering. These aspects could be relevant in the context of human PSC tumor characterization and approaching novel preclinical therapies and would deserve future investigations. Interestingly, ablation of *Pten* and *Trp53* along with *Rb1* [56,57] or Rb-family members [34] gives rise to high grade neuroendocrine tumors, supporting the role of pRb in neuroendocrine differentiation in a context of *Pten* and *Trp53* loss.

The role of *Pten* and *Trp53* in tumorigenesis has been analyzed in diverse tissues through different genetic strategies in genetically engineered mouse models. Loss of *Pten* and *Trp53* rendering adenocarcinoma progression to sarcomatoid carcinoma due to EMT transformation has been described in a murine cancer prostate model [58]. Combined inactivation of *Pten* and *Trp53* induces sarcomatoid Triple Negative Breast Cancer with enhanced features of EMT. A lower proportion of these tumors exhibit differentiated adenocarcinoma or mixed sarcomatoid plus adenocarcinoma tumors [59]. These similarities could represent a common model to explain the role of EMT in the evolution to sarcomatoid characteristics rendering a highly aggressive form of cancer. However, cell cycle regulation was found to be the driving force of liposarcoma formation or thymic lymphomas when *Pten* and *Trp53* were deleted in adipose tissue [60] or thymus [61], respectively, indicating differences in tissue susceptibility. Deregulation of mTOR (bladder) or activation of Notch signaling (smooth muscle) have also been described as mechanisms underlying tumor formation in the absence of *Pten* and *Trp53* [62,63]. Notably, basal—but not non-basal—cell type-restricted deletion of these genes in urothelial cells gives rise to muscle-invasive bladder tumors [64], allowing progression of bladder cancer in the context of inactivation of *Pten* and *Trp53*. Collectively, these data point to combined Pten and p53 exerting diverse functions/activating diverse pathways in tumor progression in a tissue-specific manner.

Mouse models of lung cancer provide critical insights into disease mechanisms [19,21,22,23], and while a number of different ADC models have been described [21,65], there is a critical need for translational PSC models that recapitulate human disease and provide opportunities for tumor characterization and pre-clinical testing. In addition, primary tumor cells isolated from these PSC tumors constitute a high valuable tool for preclinical use. It has been suggested that PSCs are transformed or dedifferentiated variants of conventional Non-Small Cell Lung Carcinoma (NSCLC) [4]. However, little is known about the biological relationships between both tumor types, and they are a current matter of debate. The Pten/p53 double-deficient mice described here develop both ADC and PSC, providing an excellent tool for this purpose, and suggests a relationship between both tumor types, supported by human-mouse transcriptomic analyses. Furthermore, as far as we know, no models of PSC have been previously reported. Although the CMV and K5-DKO mice have incomplete penetrance and long latency periods for tumor development even after naphthalene treatment, the model described here shows the role of *Pten* and *Trp53* as gene drivers of this type of tumor and the potential cells of origin from which PSC arises. Thus, the development of a murine model of PSC is significant, given the lack of models for this disease.

## 4. Materials and Methods

### 4.1. Mice and Adenoviral Infections

The *Trp53^F/F^*; *Pten^F/F^* mice were generated by breeding *Rb1^F/F^, Rbl1^−/−^, Pten^F/F^* and *Trp53^F/F^* mice [25,66] with FVB/NJ mice (purchased to the Jackson Laboratory, Strain #001800). All animal experiments were approved by the Animal Ethical Committee and conducted in compliance with the CIEMAT guidelines. Specific procedures were approved by Comunidad Autónoma de Madrid (ProEX 208/15; ProEX 111.1/21).

Ablation of *Trp53* and *Pten* in pulmonary cells was achieved by intratracheal administration of 10^8^ plaque-forming units of Ad5-CMVcre and Ad5-K5cre to 8–10-week-old mice [45]. Adenoviruses Ad5-CMVcre and custom-made Ad5-K5cre were obtained from the Viral Vector Production Unit of the Autónoma University of Barcelona [25]. As control animals, uninfected *Trp53^F/F^*; *Pten^F/F^* littermates were used. Mice were sacrificed 5 to 29 months after the adenoviral infection. Mice were sacrificed when they showed any symptom of respiratory disease or sign of illness (labored breathing, lethargy, hunched back, ruffled hair or 10–15% loss of median body weight).

### 4.2. Naphthalene Treatment

Naphthalene solution (20 mg/mL) was prepared dissolving naphthalene (Sigma-Aldrich, St. Louis, MO, USA) in corn oil (Sigma-Aldrich, St. Louis, MO, USA) by gentle rocking at room temperature for 60 min and passed through a 0.2 mm filter to remove any undissolved solute. A single dose of naphthalene was delivered to adult mice by intraperitoneal injection (200 mg naphthalene per kg body weight) three days before intratracheal administration of adenovirus. As control animals, *Trp53^F/F^*; *Pten^F/F^* littermates were given a dose of naphthalene.

### 4.3. Genotyping

Genomic DNA was isolated from *Trp53^F/F^*; *Pten^F/F^* control lungs and tumors using DNeasy Blood & Tissue Kit (Qiagen, Valencia, CA, USA). Primers sequences, amplified fragments and PCR amplification product sizes are in Table S7. *Fabpi* gene was used as loading control of samples.

### 4.4. Histology and Immunostaining/Immunohistochemistry

At necropsy, lungs were perfused with 4% formaldehyde. Samples were fixed in 4% buffered formalin and embedded in paraffin wax. Sections (5 μm) were stained with hematoxylin and eosin (H/E) for histological analysis or processed for immunohistochemistry. Immunohistochemical analyses were performed essentially as in previously described standard protocols [67,68]. Antibodies used are listed in Table S8.

### 4.5. RNA Extraction and RT-qPCR

RNA was isolated from whole mouse lungs in control mice and tumors using RNALater (Ambion Inc., Austin, TX, USA) and miRNeasy Mini Kit (Qiagen GmbH, Hilden, Germany) according to the manufacturer’s instructions (control lungs *n* = 3; ADC *n* = 5; PSC *n* = 4). Genomic DNA was eliminated from the samples by a DNase treatment (Rnase-Free Dnase Set, Qiagen GmbH, Hilden, Germany). The Omniscript RT kit (Qiagen GmbH, Hilden, Germany) and oligo dT primers were used to prepare cDNA from RNA of the mouse samples, using 2 μg of total RNA. Real-time quantitative PCR was done on a 7500 Fast Real-Time PCR system (Applied Biosystems, Foster, CA, USA) with the GoTaq qPCR Master Mix (Promega, Madison, WI, USA), using 1 μL of cDNA (as a template). Each sample was normalized using the values for the TATA binding protein gene (*Tbp*). The sequences of the specific oligonucleotides used are listed in Table S9. Discrimination between samples showing increased or decreased relative expression was made using the Mean ± SEM.

### 4.6. Western Blot Analysis

Protein extracts were obtained from control lungs, ADC and PSC tumors. Total protein extracts (50 µg) from each sample were subjected to SDS-PAGE and transferred to nitrocellulose membranes (Amersham Biosciences, Arlington Heights, IL, USA). Membranes were blocked in PBS (Phosphate-buffered saline) containing 5% BSA (bovine serum albumin) and immunodetection was perform using antibodies against phospho-Akt (Ser473) (D9E) (Cell Signalling Technology, Danvers, MA, USA), Akt (pan) (C67E7) (Cell Signalling Technology, Danvers, MA, USA) and vinculin (hVIN-1) (Sigma-Aldrich, San Luis, MO, USA). In all cases, membranes were incubated with a horseradish peroxidase (HRP)-labeled secondary antibody and detected by luminography using Immobilin Western Chemiluminescent HRP Substrate (Millipore, Burlington, MA, USA).

### 4.7. Transcriptome Analyses

Total RNA was isolated from normal lungs and tumors as described above. RNA yield and quality were determined using Agilent 2100 Bioanalyzer (RNA Integrity Number RIN > 8) (Agilent, Santa Clara, CA, USA). A total of six normal lung (from *Trp53^F/F^*; *Pten^F/F^*-uninfected mice), seven ADC and six PSC samples (Table S3) were used for microarrays experiments with Mouse Gene 2.0 ST arrays from Affymetrix (Santa Clara, CA, USA) using GeneChip WT Pico Reagent kit (Thermo Fisher Scientific, Waltham, MA, USA) following the manufacturer’s instructions. Hybridization was performed at the Oncogenomic CIEMAT-Fundación i + 12 Mix Unit in Madrid, Spain using a GeneChip Hybridization Oven 640, GeneChip Fluidics Station 450 and GeneChip 3000 7G Scanner from Affymetrix (Santa Clara, CA, USA). All the microarray data are available at Gene Expression Omnibus (GEO; http://www.ncbi.nlm.nih.gov/geo/; accession number GSE199905 (access release date 30 September 2022)). Raw data were normalized and log_2_ transformed at the gene level using the Transcriptome Analysis Console version 4.0.1 software (TAC, Affymetrix, Santa Clara, CA, USA). TAC was used to analyze variations in the transcriptome across control lung, ADC and PSC. These variations are represented in Principal Component Analyses (PCA) plots showing the distribution of the samples according to the different parameters examined (sample type, naphthalene treatment or adenoviral vector administered). TAC was also used to identify genes differentially expressed ADC and PSC versus normal lung, selecting genes with a false discovery rate (FDR) threshold of ≤0.05 and an expression fold change (FC) of ± 2. A Venn diagram (https://bioinformatics.psb.ugent.be/webtools/Venn/ (accessed on 27 May 2021)) was used to represent the significantly upregulated genes in ADC or PSC tumors versus control lung. DAVID, annotation database software (http://david.abcc.ncifcrf.gov/home.jsp (accessed on 3 June 2021)) [69], was used to identify Gene Ontology Biological Process (GOBP) functional categories of genes upregulated only in ADC, only in PSC or in both tumor types.

The web-based tool Gene Set Enrichment Analysis (GSEA, www.broadinstitute.org/gsea (accessed on 27 may 2021)) [70] was used to analyze signature enrichment. In brief, GSEA determines whether an a priori defined set of genes (gene set) shows statistically significant, concordant differences between two biological states (dataset) called phenotypes, e.g., mouse PSC tumors versus mouse ADC tumors. Hallmark gene sets and signatures for human lung ADC, SCLC and NSCLC were obtained from MSigDB (http://www.gsea-msigdb.org/gsea/msigdb/ (accessed on 23 February 2022)).

To elaborate a human lung SDC signature, gene-level, upper-quartile normalized counts converted to log2-transformed RPKM expression data were obtained from Table S2D Gillette et al. [38]. Gene expression in tumors (huLADC, *n* = 110) was compared to normal adjacent tissue (NAT, *n* = 101) using *t*-test Welch approximation. Genes with FDR < 0.001 and FC expression > 2 were selected. Additionally, we downloaded the RNAseq data from the TCGA Lung Adenocarcinoma database (https://xenabrowser.net/datapages/?dataset=TCGA.LUAD.sampleMap%2FHiSeqV2&host=https%3A%2F%2Ftcga.xenahubs.net&removeHub=https%3A%2F%2Fxena.treehouse.gi.ucsc.edu%3A443 (accessed on 23 February 2022)). Data (IlluminaHiSeq_RNASeqV2) were gene-level transcription estimates, as in log2 (x + 1) transformed RSEM normalized count. As for the Gillette dataset, gene expression in tumors (huLADC, *n* = 517) was compared to normal adjacent tissue (NAT, *n* = 59) using T-test Welch approximation. Genes with FDR < 0.001 and FC expression > 2 were selected.

### 4.8. Statistical Analyses

Comparisons between two groups were performed using Student’s unpaired t-test or Mann–Whitney test depending on the normal distribution of the data. Tumor-free survival analyses were performed using the Kaplan–Meier method and statistical differences between the two groups were tested by the log-rank test. Contingency analyses were performed using Fisher’s exact test. Statistical significance was accepted at *p* < 0.05. GraphPad Prism 6.0, 9.0 software was used.

## 5. Conclusions

This work shows that combined deletion of *Pten* and *Trp53* in mouse lung leads to the development of ADC and PSC, irrespective of the targeted cell type in which the gene alterations initially occur at least after naphthalene treatment. Naphtalene-induced epithelial airway injury prior to the inactivation of *Pten* and *Trp53* favors the development of PSC. The tumors originated show transcriptomic similarities to the human ADC reported data, supporting a relationship between both tumor types.

To our knowledge, no PSC models have been described so far. Given the paucity of human material and the lack of models for this disease, the development of a model for PSC provides a valuable tool for the understanding of the disease and the development of novel therapeutic approaches.

## Data Availability

All the microarray data are available at Gene Expression Omnibus (GEO; http://www.ncbi.nlm.nih.gov/geo/; accession number GSE199905 (access release date 30 September 2022)).

## Supplementary Materials

The following supporting information can be downloaded at: https://www.mdpi.com/article/10.3390/cancers14153671/s1, Figure S1: I Representative images of DKO mice; Figure S2: Naphthalene induced lung injury in *Trp53^F/F^*; *Pten^F/F^*-uninfected mice; Figure S3: Histology of tumors developed in DKO mice; Figure S4: Development of metastases in double-deficient mice; Figure S5: Hallmark Gene Set Enrichment Analysis (GSEA) of mouse tumors and normal lung; Figure S6: Venn diagram of human lung cancer signatures; Table S1: Lung tumors in conditional mutant *Trp53^F/F^*; *Pten^F/F^* mutant mice; Table S2: Type and number of tumors per mouse; Table S3: Samples used in the transcriptome analysis of lung, ADC and PSC; Table S4: Gene expression data of mouse lung, moADC and moPSC samples; Table S5: Genes differentially regulated in moADC tumors versus moLung and in moPSC tumors versus moLung; Table S6: Human lung ADC signatures; Table S7: Genotyping primers, amplified fragments and PCR amplification product sizes; Table S8: Primary antibodies used in the immunohistochemistry analyses; Table S9: Primers for quantitative RT-PCR analysis of gene expression; File S1: Original image of PCR and western blot.
