# Supplementary material for: Pten and p53 Loss in the Mouse Lung Causes Adenocarcinoma and Sarcomatoid Carcinoma
Source: Cancers (Basel). 2022 Jul 28;14(15):3671. doi: 10.3390/cancers14153671 (PMC9367331; doi:10.3390/cancers14153671)
Supplement: Supplementary file 1 [file cancers-14-03671-s001.zip › Lazaro et al Supplementary Figures Revised.pptx]

## Slide 1
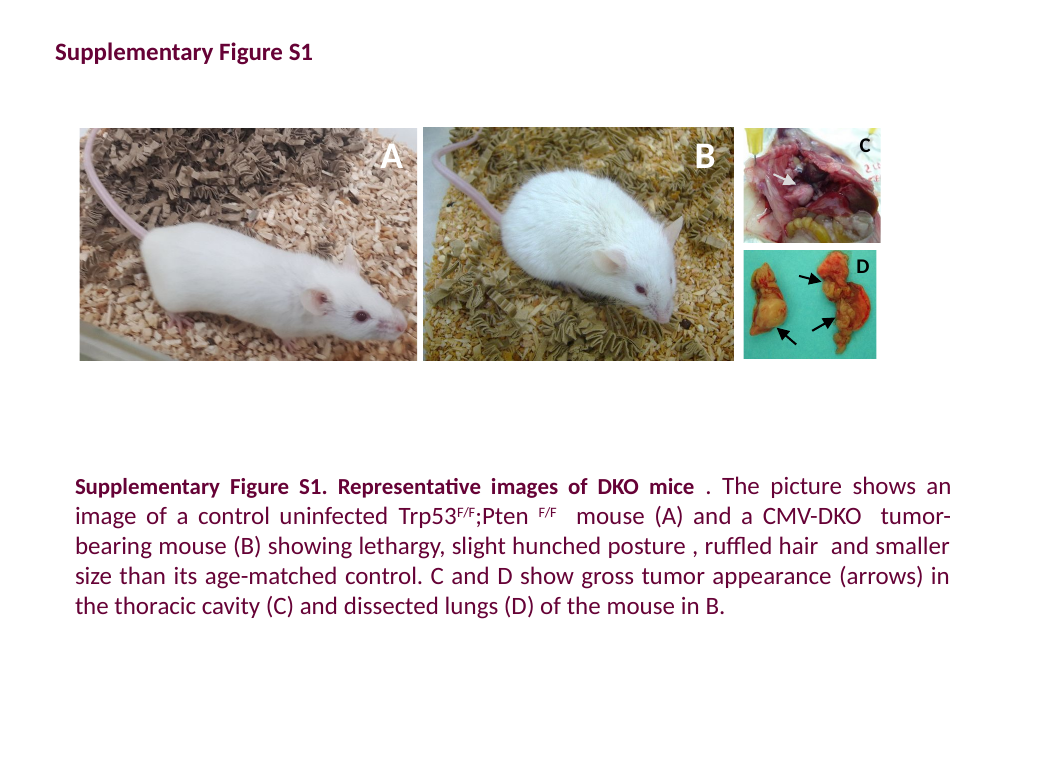

Supplementary Figure S1
C
A
B
D
Supplementary Figure S1. Representative images of DKO mice . The picture shows an image of a control uninfected Trp53F/F;Pten F/F mouse (A) and a CMV-DKO tumor-bearing mouse (B) showing lethargy, slight hunched posture , ruffled hair and smaller size than its age-matched control. C and D show gross tumor appearance (arrows) in the thoracic cavity (C) and dissected lungs (D) of the mouse in B.

## Slide 2
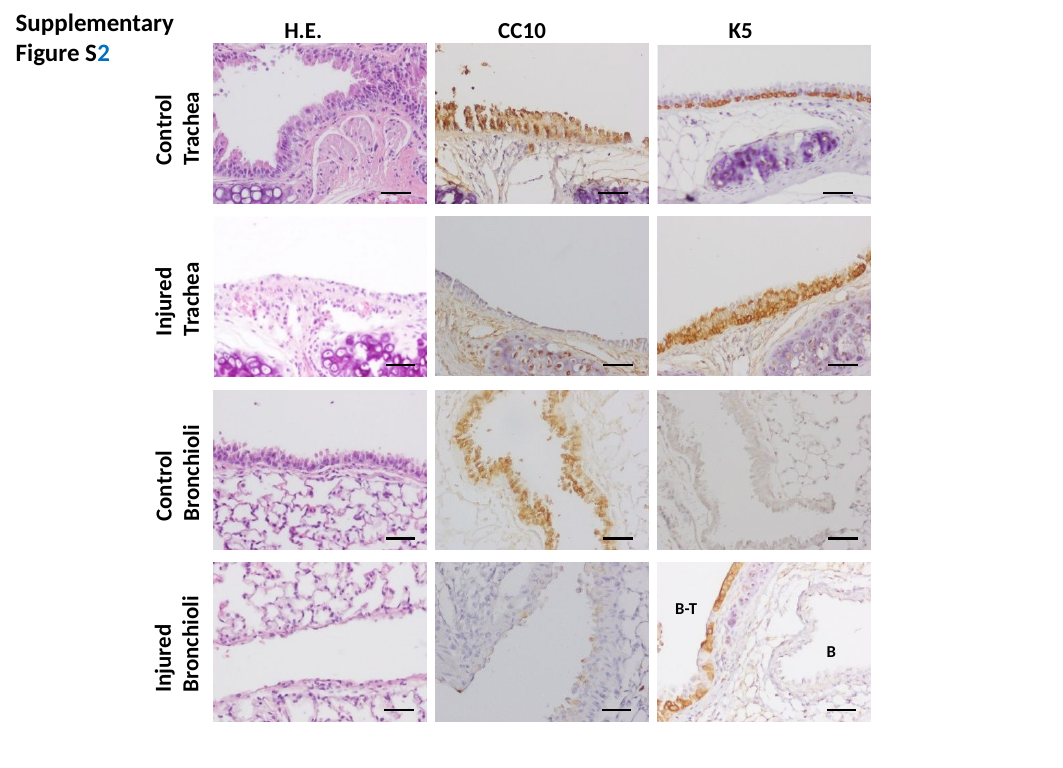

Supplementary
Figure S2
H.E.
CC10
K5
Control Trachea
Injured Trachea
Control Bronchioli
B-T
Injured Bronchioli
B

## Slide 3
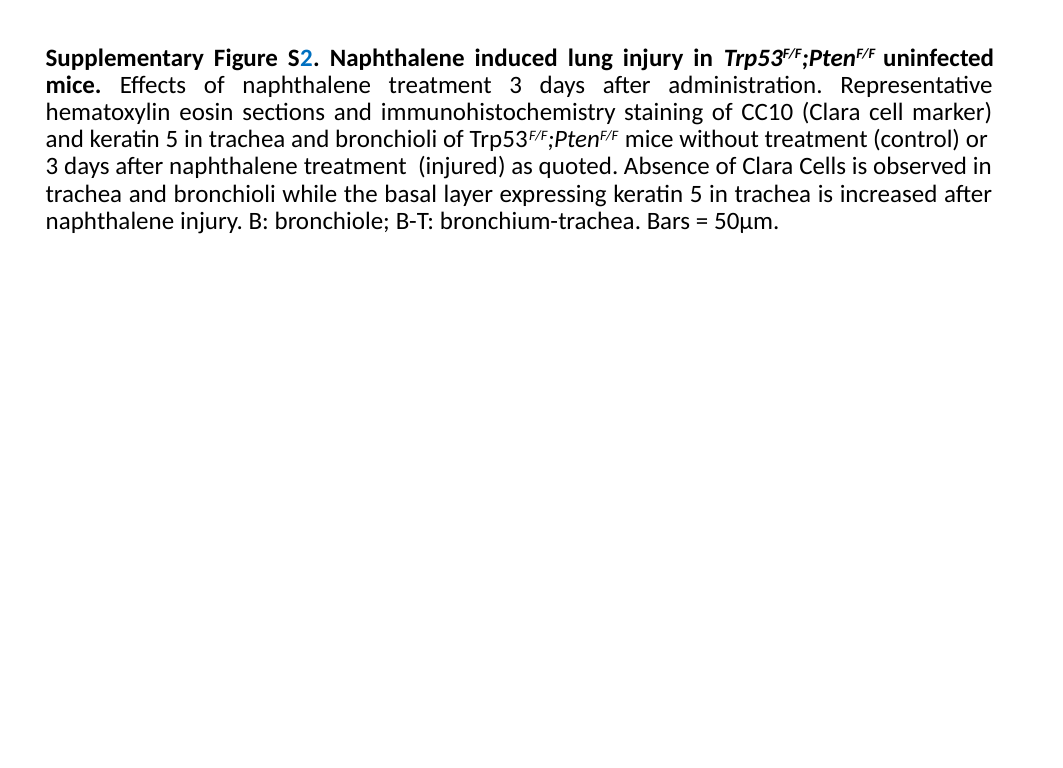

Supplementary Figure S2. Naphthalene induced lung injury in Trp53F/F;PtenF/F uninfected mice. Effects of naphthalene treatment 3 days after administration. Representative hematoxylin eosin sections and immunohistochemistry staining of CC10 (Clara cell marker) and keratin 5 in trachea and bronchioli of Trp53F/F;PtenF/F mice without treatment (control) or 3 days after naphthalene treatment (injured) as quoted. Absence of Clara Cells is observed in trachea and bronchioli while the basal layer expressing keratin 5 in trachea is increased after naphthalene injury. B: bronchiole; B-T: bronchium-trachea. Bars = 50µm.

## Slide 4
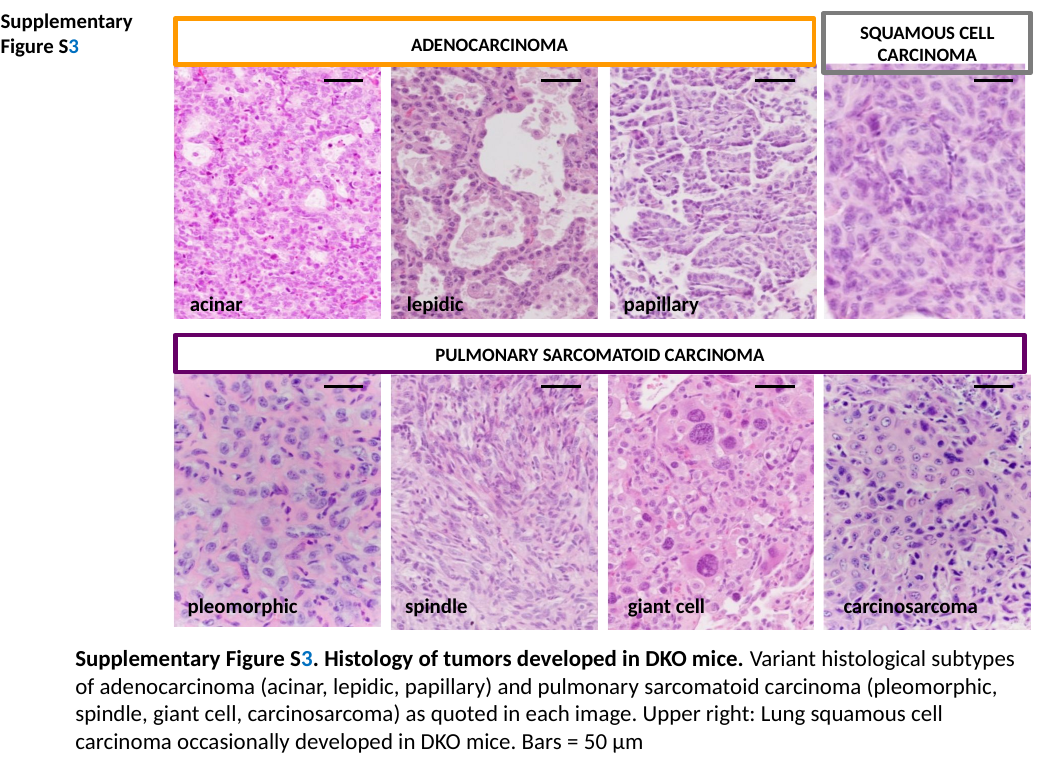

Supplementary
Figure S3
SQUAMOUS CELL CARCINOMA
ADENOCARCINOMA
acinar
lepidic
papillary
PULMONARY SARCOMATOID CARCINOMA
pleomorphic
 spindle
giant cell
carcinosarcoma
Supplementary Figure S3. Histology of tumors developed in DKO mice. Variant histological subtypes of adenocarcinoma (acinar, lepidic, papillary) and pulmonary sarcomatoid carcinoma (pleomorphic, spindle, giant cell, carcinosarcoma) as quoted in each image. Upper right: Lung squamous cell carcinoma occasionally developed in DKO mice. Bars = 50 µm

## Slide 5
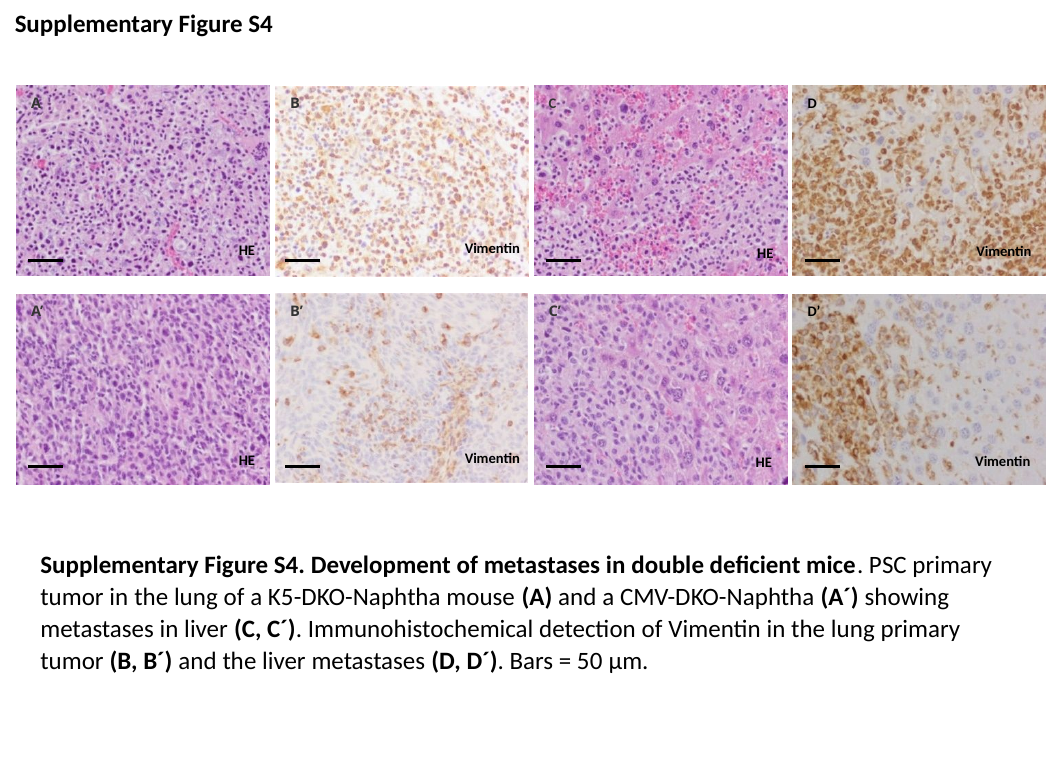

Supplementary Figure S4
A
C
D
B
Vimentin
HE
Vimentin
HE
A’
B’
C’
D’
Vimentin
HE
Vimentin
HE
Supplementary Figure S4. Development of metastases in double deficient mice. PSC primary tumor in the lung of a K5-DKO-Naphtha mouse (A) and a CMV-DKO-Naphtha (A´) showing metastases in liver (C, C´). Immunohistochemical detection of Vimentin in the lung primary tumor (B, B´) and the liver metastases (D, D´). Bars = 50 μm.

## Slide 6
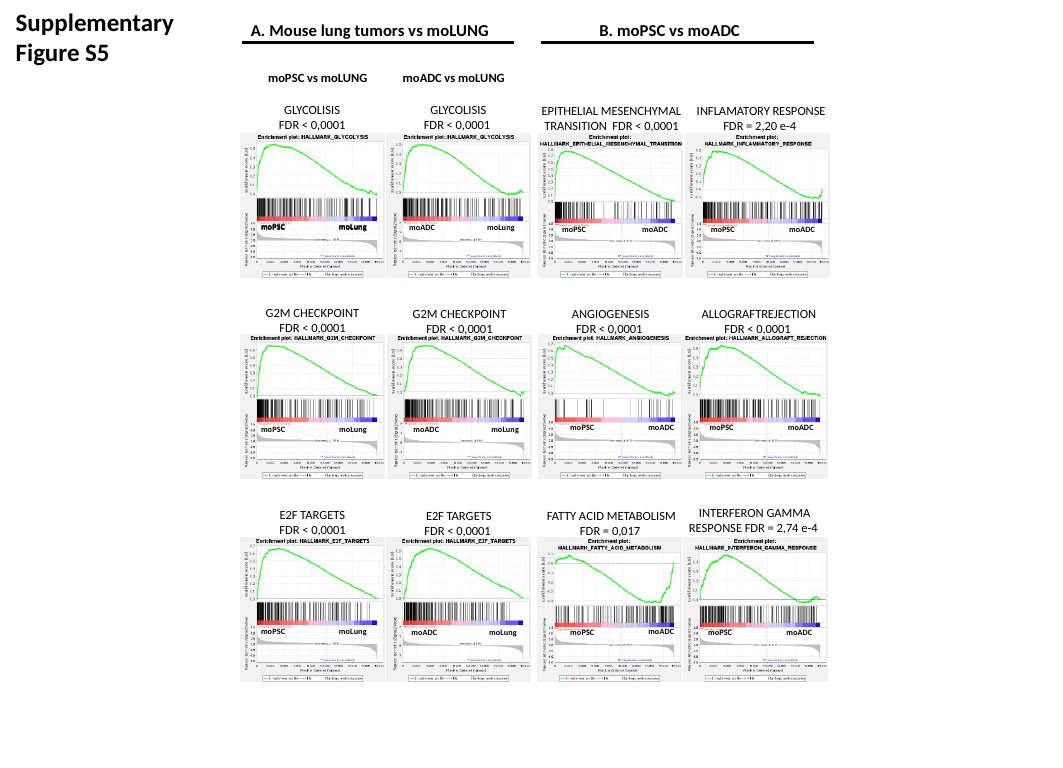

Supplementary
Figure S5
A. Mouse lung tumors vs moLUNG
B. moPSC vs moADC
moPSC vs moLUNG
moADC vs moLUNG
GLYCOLISIS
FDR < 0,0001
GLYCOLISIS
FDR < 0,0001
EPITHELIAL MESENCHYMAL TRANSITION FDR < 0,0001
INFLAMATORY RESPONSE
FDR = 2,20 e-4
moPSC
moLung
moPSC
moLung
moADC
moLung
moPSC
moADC
moADC
moPSC
G2M CHECKPOINT
FDR < 0,0001
G2M CHECKPOINT
FDR < 0,0001
ANGIOGENESIS
FDR < 0,0001
ALLOGRAFTREJECTION
FDR < 0,0001
moADC
moADC
moPSC
moPSC
moPSC
moLung
moADC
moLung
INTERFERON GAMMA RESPONSE FDR = 2,74 e-4
E2F TARGETS
FDR < 0,0001
FATTY ACID METABOLISM
FDR = 0,017
E2F TARGETS
FDR < 0,0001
moADC
moPSC
moLung
moPSC
moADC
moLung
moADC
moPSC

## Slide 7
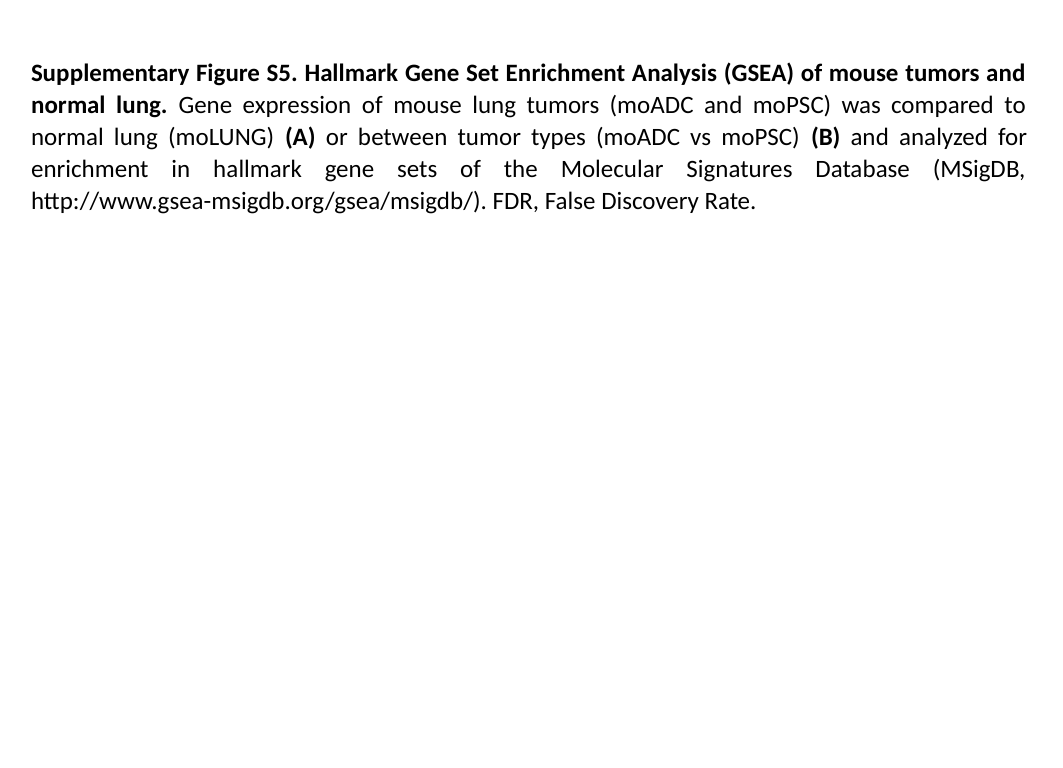

Supplementary Figure S5. Hallmark Gene Set Enrichment Analysis (GSEA) of mouse tumors and normal lung. Gene expression of mouse lung tumors (moADC and moPSC) was compared to normal lung (moLUNG) (A) or between tumor types (moADC vs moPSC) (B) and analyzed for enrichment in hallmark gene sets of the Molecular Signatures Database (MSigDB, http://www.gsea-msigdb.org/gsea/msigdb/). FDR, False Discovery Rate.

## Slide 8
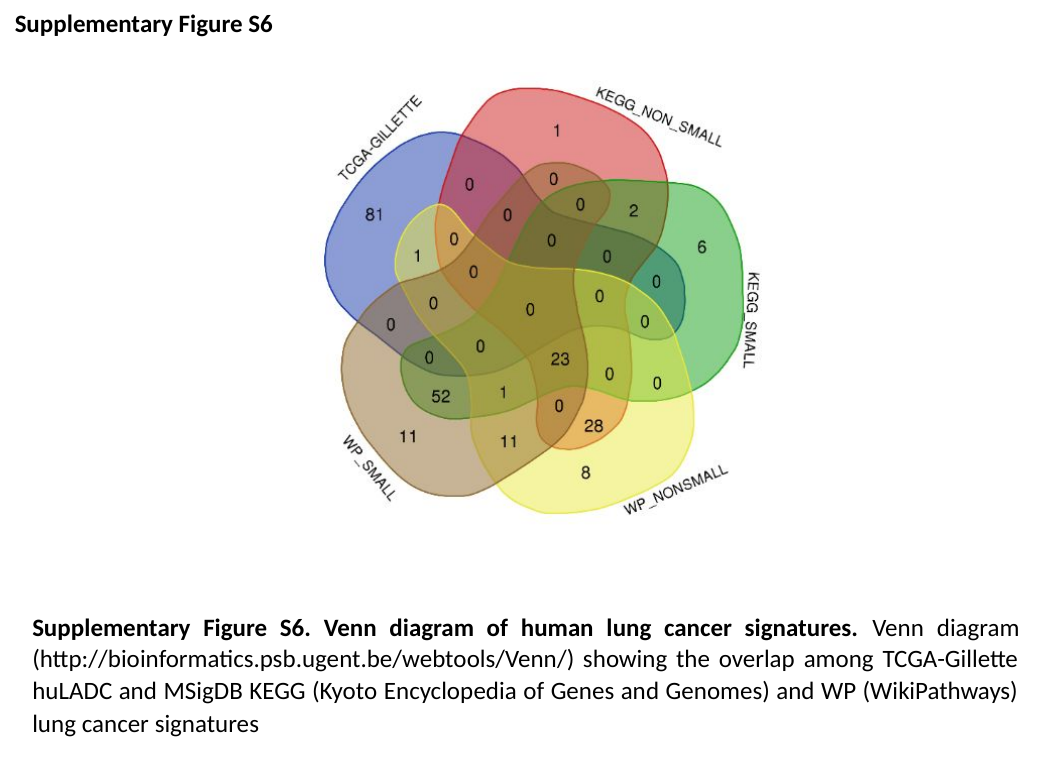

Supplementary Figure S6
Supplementary Figure S6. Venn diagram of human lung cancer signatures. Venn diagram (http://bioinformatics.psb.ugent.be/webtools/Venn/) showing the overlap among TCGA-Gillette huLADC and MSigDB KEGG (Kyoto Encyclopedia of Genes and Genomes) and WP (WikiPathways) lung cancer signatures
