# Supplementary material for: Pten and p53 Loss in the Mouse Lung Causes Adenocarcinoma and Sarcomatoid Carcinoma
Source: Cancers (Basel). 2022 Jul 28;14(15):3671. doi: 10.3390/cancers14153671 (PMC9367331; doi:10.3390/cancers14153671)
Supplement: Supplementary file 1 [file cancers-14-03671-s001.zip › Lazaro et al Supplementary Tables 1,3,7,8,9 Revised.pdf]

Supplementary Table S1. Lung tumors in conditional mutant $Trp53^{F/F}$; $Pten^{F/F}$ mutant mice

Group	Total # of mice	# Mice with described pathology/ # total of mice	Major lung tumor type	# of sacrificed mice (months post-infection)					
				5-8	9-13	14-17	18-21	22-25	26-29
**CMV-DKO**	18	11/18	No lesions	1	2	2	3	3	
		5/18	ADC	1		2	2		
		2/18	PSC+ADC				1	1	
**K5-DKO**	19	14/19	No lesions		3	4	2	3	2
		5/19	ADC				2	2	1
**CMV-DKO -Naphtha**	22	9/22	No lesions	2	5	1	1		
		4/22	ADC		3		1		
		4/22	PSC	1	2	1			
		3/22	PSC+ADC	1			2		
		1/22	PSC+ADC+ SCC					1	
		1/22	SCC				1		
**K5-DKO -Naphtha**	19	10/19	No lesions		1	1	2	4	2
		4/19	ADC				3	1	
		1/19	ADC+SCC					1	
		1/19	PSC					1	
		2/19	PSC+ADC		1			1	
		1/19	PSC+ADC+ SCC					1	

Abbreviations: ADC: Adenocarcinoma; PSC: Pulmonary Sarcomatoid Carcinoma; SCC: Squamous Cell Carcinoma

Supplementary Table S3. Samples used in the transcriptome analysis of lung, ADC, PSC.

HISTOPATHOLOGY GROUP	SAMPLE	ADENOVIRUS	NAPHTHALENE
LUNG	LUNG 1	NO	NO
	LUNG 2	NO	NO
	LUNG 3	NO	NO
	LUNG 4	NO	NO
	LUNG 5	NO	NO
	LUNG 6	NO	NO
ADENOCARCINOMA (ADC)	ADC DKO 1	CMV	NO
	ADC DKO 2	CMV	NO
	ADC DKO 3	CMV	NO
	ADC DKO 4	CMV	YES
	ADC DKO 5	K5	YES
	ADC DKO 6	K5	YES
	ADC DKO 7	K5	NO
PULMONARY SARCOMATOID CARCINOMA (PSC)	PSC DKO 1	CMV	YES
	PSC DKO 2	CMV	YES
	PSC DKO 3	CMV	YES
	PSC DKO 4	CMV	YES
	PSC DKO 5	CMV	NO
	PSC DKO 6	K5	YES

Supplementary Table S7. Genotyping Primers, amplified fragments and PCR amplification product sizes.

Gene	Forward (F) or Reverse (R) Primer	Primer Sequence (5' → 3')	Amplified Fragment	Product size pb)
p53	Forward	AAGGGGTATGAGGGACAAGG	***p53**^{wt}*	431
	Reverse	GAAGACAGAAAAGGGGAGGG	***p53**^{flox}*	584
p53	Forward	CACAAAAAACAGGTTAAACCCAG	***p53**$^{\Delta}$*	612
	Reverse	GAAGACAGAAAAGGGGAGGG		
Pten	Forward	CCATTACCTAGTAAAGCAAG	***Pten**^{wt}*	230
	Reverse	TGCAAAGAATCTTGGTGTTAC	***Pten**^{flox}*	276
Pten	Forward	TGGCATAAAGTTAGGAAAAGATG	***Piten**$^{\Delta}$*	330
	Reverse	TGCAAAGAATCTTGGTGTTAC		
Fabpi	Forward	TGGACAGGACTGGACCTCTGCTTTCCTAGA	***Fabpi**^{wt}*	194
	Reverse	TAGAGCTTTGCCACATCACAGGTCATTCAG		

Supplementary Table S8. Primary Antibodies used in the Immunohistochemistry analyses

Primary Antibodies used in Immunohistochemistry		
**Antibody Name**	**Manufacturer and location**	**Dilution**
**anti-Clara cell secretory protein, CC10 (T-18)**	Santa Cruz Biotechnology, Dallas, TX, USA	1:200
**anti-Clara cell secretory protein ,CC10 (H-75)**	Santa Cruz Biotechnology, Dallas, TX, USA	1:200
**anti-K5**	Biolegend, San Diego, CA, USA	1:500
**anti- phosphatase and tensin homolog (PTEN)**	Cell Signalling, Danvers, MA, USA	1:100
**Anti-p53**	Novocastra, Leica Biosystems, Newcastle, UK	1:500
**anti-thyroid transcription factor-1 (TTF-1)**	Abcam-Epitomics, Cambridge, UK	1:200
**anti-keratin 7 (clone RCK105)**	Abcam, Cambridge, UK	1:500
**anti-pan Cytokeratin antibody (AE1-AE3)**	Abcam, Cambridge, UK	1:40
**anti-vimentin**	Abcam, Cambridge, UK	1:100
**anti-keratin 8 (clone TROMA-I) , K8**	DSHB, Kosciusko, MS, USA	1:10
**anti-p63 (clone 4A4)**	Abcam, Cambridge, UK	1:100
**MASH1 anti-ASCL1**	Abcam, Cambridge, UK	1:200
**anti-Calcitonin Gene Related Peptide (CGRP)**	Sigma-Aldrich, St Louis, MO	1:1000
**anti-Phospho-AKT (Ser473)**	Cell Signaling Technology, Denvers, MA, USA	1:100
**anti-mTOR**	Abcam, Cambridge, UK	1:400
**anti-Phospho70S6K (Thr421/Ser424) (P-70S6K)**	Cell Signaling Technology, Denvers, MA, USA	1:100
**anti-Phospho-S6 (Ser235/236) (P-S6)**	Cell Signaling Technology, Denvers, MA, USA	1:100
**anti-E-cadherin (E-cad)**	BD Transduction Lab, NJ, USA	1:50
**anti-Snail**	Cell Signaling Technology, Denvers, MA, USA	1:100

Supplementary Table S9. Primers for quantitative RT-PCR analysis of gene expression.

Primers for quantitative RT-PCR analysis of gene expression		
**Gene**	**Forward primer sequence (F) 5' -> 3'**	**Reverse primer sequence (R) 5' -> 3'**
Cdh1	ATCCTCGCCCTGCTGATT	ACCACCGTTCTCCTCCGTA
Tbp	GGGAGAATCATGGACCAGAA	GATGGGAATTCCAGGAGTCA
Snai1	CTTGTGTCTGCACGACCTGT	GGAGCAGGAGAATGGCTTC
Snai2	CATTGCCTTGTGTCTGCAAG	CATTGCCTTGTGTCTGCAAG
Vim	CCAACCTTTTCTTCCCTGAAC	TTGAGTGGGTGTCAACCAGA
Pten	GAA AGG GAC GGA CTG GTG TA	TAG GGC CTC TTG TGC CTT TA
Trp53	GCC CAT GCT ACA GAG GAG TC	AGA CTG GCC CTT CTT GGT CT
