# Supplementary figures and images for: Pten and p53 Loss in the Mouse Lung Causes Adenocarcinoma and Sarcomatoid Carcinoma
Source: Cancers (Basel). 2022 Jul 28;14(15):3671. doi: 10.3390/cancers14153671 (PMC9367331; doi:10.3390/cancers14153671)

Original PCR figure for figure 3

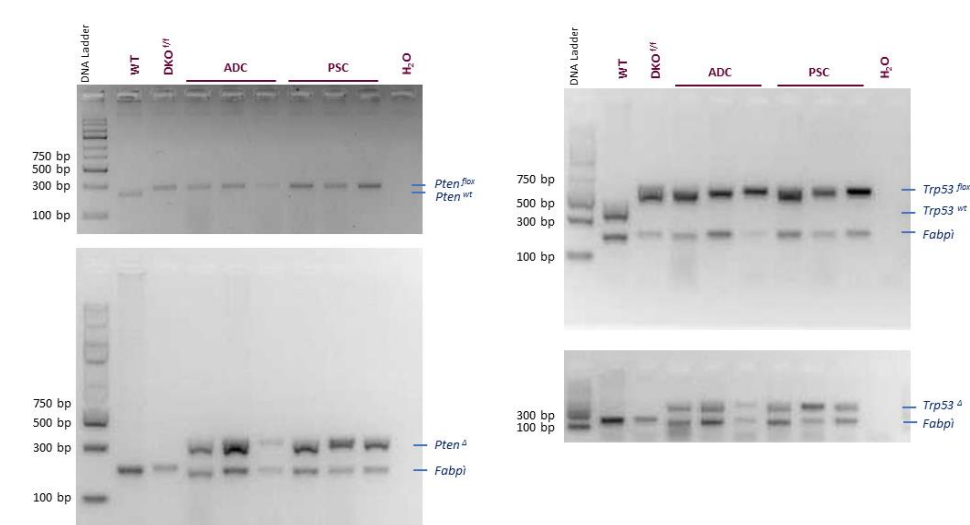

Original WB figure for figure 4

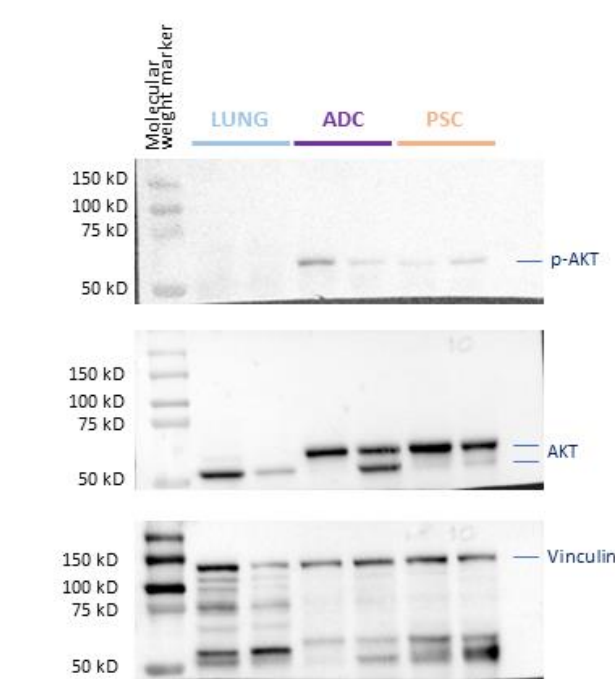

Supplement: Supplementary file 1 [file cancers-14-03671-s001.zip › File S1 revised lazaro et al Cancers 1827993.pdf]
